# Supplementary material for: Developing Cardio-Oncology Programs in the New Era: Beyond Ventricular Dysfunction Due to Cancer Treatments
Source: Cancers (Basel). 2023 Dec 18;15(24):5885. doi: 10.3390/cancers15245885 (PMC10742309; doi:10.3390/cancers15245885)
Supplement: Supplementary file 1 [file cancers-15-05885-s001.zip › cancers-2764907-supplementary.pdf]

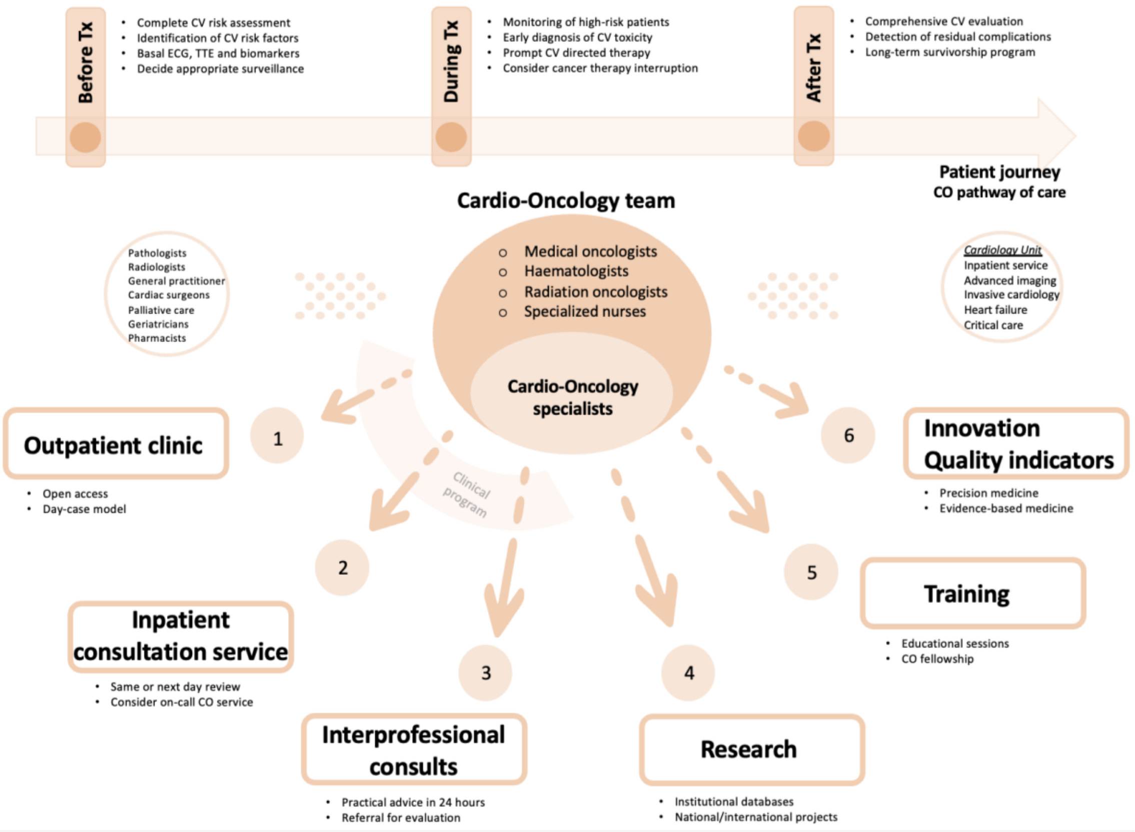

Supplementary Figure S1: Clinical care pathway and main components of a Cardio-Oncology program. Abbreviations: CO, Cardio-Oncology; CV, Cardiovascular; ECG, Electrocardiogram; TTE, Transthoracic echocardiography; Tx, Treatment.
